# *Bacillus coagulans* idrc019 Attenuates Irritable Bowel Syndrome by Revealing Multimodal Protective Mechanisms

**DOI:** 10.3390/microorganisms14030701

**Published:** 2026-03-20

**Authors:** Yi-Wei Jin, Feng Chen, Jiang Cao

**Affiliations:** Innovative Drug Research Center, College of Life Sciences and Medicine, Key Laboratory of Plant Secondary Metabolism and Regulation of Zhejiang Province, Zhejiang Sci-Tech University, Hangzhou 310018, China; 2024210901038@mails.zstu.edu.cn (Y.-W.J.); 2025220902003@mail.zstu.edu.cn (F.C.)

**Keywords:** probiotic bacteria, irritable bowel syndrome, gut microbiota, metabolomics

## Abstract

*Bacillus coagulans* has attracted widespread attention in the treatment of irritable bowel syndrome due to its multiple probiotic functions, yet its specific molecular mechanisms remain unclear, and the efficacy of probiotics exhibits significant strain specificity, posing a key bottleneck for practical application. To address this, this study obtained a bile salt-tolerant *B. coaguans* idrc019 through *in vitro* screening. This strain demonstrated strong survival and germination in simulated gut conditions, supporting effective intestinal colonization. Further evaluation in an IBS animal model revealed that idrc019 alleviated visceral hypersensitivity and colonic inflammation in a dose-dependent manner. Through enhanced intestinal barrier integrity, microbiota modulation (e.g., *Actinobacteria* restoration), and elevated metabolites (e.g., kynurenine), the strain exerted IBS-alleviating effects via synchronized immune, microbial, and metabolic regulation. Our findings offer a mechanistically grounded probiotic candidate, underscore functional screening as a critical strategy, and pave the way for clinical application.

## 1. Introduction

Irritable bowel syndrome (IBS) is a common functional gastrointestinal disorder with a high global prevalence. It is characterized by recurrent abdominal pain, bloating, and altered bowel habits (such as diarrhea, constipation, or a mixed pattern), which significantly impair patients’ quality of life [1]. The pathophysiological mechanisms of IBS are complex, involving interactions among multiple factors, including visceral hypersensitivity, abnormal intestinal motility, gut–brain axis dysregulation, low-grade intestinal inflammation, gut microbiota disturbances, and immune activation [2]. Current management strategies for IBS focus on symptom relief and improving quality of life, often employing a hierarchical and individualized approach. This includes dietary modifications (e.g., low-FODMAP diet), psychological interventions (e.g., cognitive behavioral therapy), and pharmacological treatments (e.g., antispasmodics, antidiarrheals, laxatives, and neuromodulators) [3]. However, these therapies have notable limitations. For instance, most drugs targeted only single symptoms with uncertain long-term efficacy and potential side effects [4]. Therefore, there is a pressing need to explore safe, effective therapeutic strategies capable of multi-target intervention in the IBS pathophysiological process.

In this context, probiotic therapy has garnered significant attention due to its pleiotropic effects, such as modulating gut microbiota, enhancing barrier function, regulating immunity, and influencing neural activity [5]. Substantial clinical and preclinical evidence indicates that various probiotics (e.g., lactobacilli and *Bifidobacterium*) can alleviate IBS symptoms through these mechanisms [6]. *Bacillus coagulans* (*B. coagulans*) is a spore-forming, Gram-positive bacterium that possesses acid-producing properties and strong stress resistance. Its spore structure effectively resists gastrointestinal challenges like gastric acid and bile salts, ensuring sufficient viable bacteria reach the intestine [7]. Upon germination and colonization, it exerts probiotic actions, including modulation of gut microbiota composition, inhibition of pathogens, reinforcement of the intestinal barrier, and regulation of local and systemic immune responses, thereby demonstrating potential in alleviating IBS-related symptoms. However, the efficacy of probiotics is markedly strain-specific, meaning different strains within the same species can exhibit significantly different beneficial effects [8]. Our preliminary research suggests that the variation in probiotic functionality among *B. coagulans* strains may be closely related to their bile salt tolerance [9]. Bile salts are major antimicrobial agents in the intestine; strains tolerant to bile salts can better survive, germinate, and proliferate within the gut, ensuring the full expression of their probiotic functions [10]. Consequently, screening for specific strains with excellent bile salt tolerance and empirically validated efficacy is a prerequisite for developing effective *B. coagulans*-based probiotic products.

Despite the promising application prospects of *B. coagulans*, its specific molecular mechanisms in alleviating IBS remain insufficiently elucidated. Existing studies have primarily focused on phenotypic observations, while systematic analysis of how it coordinately exerts effects through multiple pathways, such as modulating intestinal inflammation, improving microbial structure, and influencing host metabolism (e.g., tryptophan metabolism, short-chain fatty acid production), is still lacking [11,12]. Furthermore, its dose–response relationship is unclear, which hinders standardized application and consistent therapeutic outcomes.

Based on the aforementioned background, this study first screened the bile salt-tolerant strain *B. coagulans* idrc019 (Source: human feces, Jining, Shandong, China; Sample ID: FSDJN1M2; Year: 2024) through in vitro simulation of the gastrointestinal environment and evaluated its survival and germination capabilities under simulated digestive stress. Subsequently, using an IBS animal model, we systematically investigated the mechanisms of action of this strain from multiple dimensions, including intestinal barrier integrity, inflammatory cytokine expression, gut microbiota structure, and metabolomic profiles. The dose-dependent effects were also clarified. This work aims to provide both a promising strain and mechanistic insights for developing targeted probiotic formulations against IBS.

## 2. Materials and Methods

### 2.1. Chemicals and Materials

All chemical reagents, including ox bile salts, DEPC water, absolute ethanol, glucose, hydrolyzed casein, yeast extract, soy peptone, ferrous sulfate heptahydrate, manganese sulfate monohydrate, anhydrous calcium chloride, sodium chloride, anhydrous disodium hydrogen phosphate, and anhydrous dipotassium hydrogen phosphate, were purchased from Shanghai National Medicines Co., Ltd., Shanghai, China. The mouse colon cytokine ELISA kits (IL-1β, IL-6, IL-10, and TNF-α) were obtained from R&D Systems (Shanghai) Biotech Co., Ltd., Shanghai, China. The mouse colon Occludin membrane protein ELISA kit was acquired from Nanjing Senbeijia Biotechnology Co., Ltd., Nanjing, China. ELISA kits for measuring mouse gastrointestinal mucosal sensory nerve markers (mast cell tryptase, PAR-2, and serum corticosterone) were supplied by Shanghai Enzyme-linked Biotechnology Co., Ltd., Shanghai, China. The mouse fecal genomic DNA extraction kit (FastDNA^®^ Spin Kit for Feces) was purchased from MP Biomedicals (Irvine, CA, USA). The BCA protein concentration assay kit for mouse colon tissue was procured from Beyotime Biotechnology (Shanghai, China). The DNA gel/PCR purification kit (DNA Gel/PCR Purification Miniprep Kit) was obtained from Bio-Wit Medical Technologies (Hangzhou, China). The microbial strains used in this study were obtained from the following sources: a total of 50 *B. coagulans* strains were provided by the Innovative Drug Research Center at Zhejiang Sci-Tech University, China (Table S1); *B. coagulans* ATCC 7050, *Lacticaseibacillus rhamnosus* GG, and *Citrobacter rodentium* (*C. rodentium*) DSM100 were acquired from the Guangdong Microbial Culture Center, Guangzhou, China. Vegetative cells of *B. coagulans* were cultured aerobically in MRS broth at 37 °C for 24 h and successively activated three times prior to experimental use.

### 2.2. Detection of the Survival Rate of B. coagulans Strains In Vitro

The effect of simulated gastrointestinal (GI) stress on spore viability was assessed following established methods with modifications [13]. Spores were subjected to sequential digestion phases: gastric (pepsin in NaCl, pH 3.0, 37 °C, 3 h, 50 rpm), and intestinal (ox bile and pancreatin with Oxyrase^®^, Oxyrase, Inc., Mansfield, TX, USA, pH 7.0, 37 °C, 24 h). After each phase, samples were collected and viable spores of *B. coagulans* were enumerated. Growth was quantified by measuring OD_600_ after 24 h fermentation, with MRS medium as 100% growth control.

In preliminary experiments, we simultaneously measured the OD_600_ values and performed plate counts (CFU/mL) of the test strain at different growth stages, with uninoculated MRS broth included in each experiment as a blank control for spectrophotometer zeroing. A standard OD_600_-CFU curve for the specific strain in MRS broth used in this study was thus established as previously described [13]. The bacterial concentrations at key time points reported in the main text were derived from this standard curve or verified by direct plate counting. Experiments were performed in triplicate across three independent runs, and results are expressed as percentage growth relative to the MRS control. *B. coagulans* idrc019 was selected for further functional studies.

### 2.3. Animal Experiment Design

Male C57BL mice (6–8 weeks, 22 ± 0.2 g) obtained from Zhejiang Vital River Laboratory Animal Technology Co., Ltd., Jiaxing, China. (Production License No. SCXK (Zhejiang) 2019-0001; Use License No. SYXK (Zhejiang) 2021-0001) were utilized in this study. All animal studies and procedures were conducted in accordance with the guidelines and regulations approved by the Institutional Animal Research Committee of the Zhejiang Sci-tech University (ZSTU20251026-3) and the National Research Council’s Guide for the Care and Use of Laboratory Animals (IACUC No. 3590).

Mice were randomized by body weight and age (6–8 weeks) into 5 groups of 5 mice each, and housed separately by group. The mice were acclimatized for one week prior to the experiment under the same housing conditions (25 ± 1 °C, 50–60% relative humidity, 12 h light/dark cycle) with free access to food and water.

The experimental protocol, which spanned 31 days, was adapted from an established methodology described in prior work (Table 1) [9,14]. *C. rodentium* DBS100 was used to establish a post-infectious IBS model, as it induces transient colitis followed by persistent visceral hypersensitivity, effectively mimicking post-infectious IBS pathophysiology. Bacteria were grown in LB medium at 37 °C for 20 h, harvested by centrifugation at 4 °C, washed, and resuspended in sterile saline. Concentration was determined by OD_600_ against a standard curve. Mice were inoculated by oral gavage with 1.2 × 10^10^ CFU/0.2 mL per mouse. Diarrhea was monitored for 24–48 h; if absent, a second identical gavage was given. Following confirmation of infection, the water avoidance stress (WAS) protocol was applied after the recovery period to induce a persistent IBS-like phenotype.

Upon completion of the trial, fresh fecal samples were collected and immediately preserved at −80 °C. Euthanasia was performed using isoflurane anesthesia. Blood samples were centrifuged to isolate serum, which was subsequently frozen at −80 °C for further analysis. Colon tissues were divided into two segments: one was fixed with 4% paraformaldehyde for 24 h for histological examination, and the remaining segment was flash-frozen with as follows: the tissue was placed in a sterile microcentrifuge tube, which was then immersed in liquid nitrogen for 2 min. Afterwards, the sample was stored at −80 °C until analysis.

### 2.4. Evaluation of the Efficacy of B. coagulans in Alleviating IBS

Visceral sensitivity was assessed by measuring the Abdominal Withdrawal Reflex (AWR) in response to colorectal distension, with the pain threshold defined as the pressure that elicited abdomen lifting. Visceral sensitivity was assessed by colorectal distension. Prior to the experiment, the latex balloon used for distension was calibrated in vitro with a pressure transducer to ensure accurate and reproducible pressure application. For three consecutive days before testing, mice were acclimated for 30 min daily in the same restrainer used during the experiment to minimize stress. During the test, the calibrated balloon was gently inserted approximately 2 cm into the distal colon and secured. Colorectal distension was performed in a stepwise manner with graded volumes: 0, 0.1, 0.2, and 0.3 mL. Each volume was applied twice with a 4 min interval between measurements.

The AWR was scored on a standardized scale: 0 indicated no behavioral response to distension; 1 represented a brief head movement at stimulus onset without abdominal contraction; 2 denoted mild abdominal muscle contraction without lifting of the abdomen; 3 signified strong abdominal contraction with the abdomen lifted off the platform; and 4 corresponded to body arching and lifting of the pelvis. The final AWR score for each mouse at each pressure stimulus was calculated as the average of the scores assigned by the three experimenters. Prior to the experiment, all experimenters had undergone standardized training to achieve high inter-rater reliability (Kappa coefficient > 0.8). In cases where individual scores showed substantial discrepancy the data point was re-evaluated by all experimenters through joint review of the video recording until a consensus was reached.

Colon tissue samples (approximately 1 cm in length) were aseptically collected from the distal colon and immediately fixed in 4% paraformaldehyde for 24 h at room temperature [12]. Subsequently, the tissues were processed for embedding, sectioning, and staining according to standard histological protocols. Pathological alterations, including congestion, hemorrhage, edema, necrosis, hyperplasia, fibrosis, and inflammatory cell infiltration, were examined under a microscope by observers blinded to the experimental groups. Distinct pathological differences between samples were documented, and representative lesions were photographed and marked with arrows for clarity. A blinded semi-quantitative scoring procedure: three researchers, blinded to the experimental groups, independently scored colon sections using established histological criteria. The parameters included the degree of inflammatory cell infiltration, scored on a traditional 3-point scale where 0 indicates none and 3 indicates severe infiltration [9].

### 2.5. Measurement of Intestinal Immune Regulation Indicators

Colon tissue (0.1 g) was weighed accurately, homogenized in ice-cold sterile saline, and subjected to centrifugation (3000× *g*, 4 °C, 5 min), and the supernatant was collected. Levels of interleukin (IL)-1β, IL-6, IL-10, and tumor necrosis factor-alpha (TNF-α) were measured using corresponding assay kits (R&D Systems China Co., Ltd., Shanghai, China).

### 2.6. Measurement of Intestinal Barrier Function Indicators

Colon tissue (0.1 g) was weighed accurately, homogenized in ice-cold sterile saline, and subjected to centrifugation (3000× *g*, 4 °C, 5 min) to collect the supernatant. The expression level of Occludin was determined using a specific ELISA kit (SenBeiJia Biological Technology Ltd., Nanjing, China).

### 2.7. Measurement of Gastrointestinal Sensory Nerve-Related Indicators

According to a previous study, colon tissue (0.1 g) was homogenized in ice-cold sterile saline, and subjected to centrifugation (3000× *g*, 4 °C, 5 min), and the supernatant was collected. The content of mast cell tryptase, expression of protease-activated receptor 2 (PAR-2), and serum corticosterone level were measured using appropriate kits (Shanghai Enzyme-linked Biotechnology Co., Ltd., Shanghai, China).

### 2.8. Fecal Microbiota Analysis

Fecal DNA was extracted using a DNA extraction kit. The V3-V4 region of the 16S rRNA gene was amplified using primers 341F/806R (341F: CCTAYGGGRBGCASCAG; 806R: GGACTACNNGGGTATCTAAT). The PCR program was: 95 °C for 7 min; 30 cycles of 95 °C for 30 s, 50 °C for 30 s, and 72 °C for 50 s; final extension at 72 °C for 10 min and holding at 12 °C for 10 min. After gel recovery, DNA concentration was measured with an Illumina NovaSeq 6000 platform (2 × 250 bp), Illumina Inc., San Diego, CA, USA. DNA concentration was determined using a Qubit Fluorometer. Libraries were constructed with the NEXTFLEX^®^ 16S V4 Amplicon-Seq Kit, Bioo Scientific (a PerkinElmer company, Austin, TX, USA). Bioinformatic analysis was conducted in QIIME2 (Version 2023.5): reads were processed with DADA2 to generate ASVs, which were taxonomically classified against the SILVA 138 database; subsequent analyses were performed in R using the phyloseq (Version 1.44.0) and vegan (Version 2.6-4) packages.

### 2.9. Fecal Metabolomics Analysis

Fecal metabolite profiling was performed using an untargeted strategy based on liquid chromatography–tandem mass spectrometry (LC–MS/MS) to comprehensively screen for microbiota-derived and host metabolites, including short-chain fatty acids [15]. Briefly, approximately 100 mg of feces was homogenized with 400 μL of pre-cooled methanol containing stable isotope-labeled internal standards (for process correction) using grinding beads. After centrifugation, the supernatant was collected, evaporated under a stream of nitrogen, and reconstituted in 100 μL of methanol: water (8:2, *v*/*v*). A quality control (QC) sample was prepared by pooling equal volumes of all sample extracts and was injected at regular intervals throughout the analytical sequence to monitor system stability. Chromatographic separation was carried out on a Waters Acquity I-Class PLUS system equipped with an HSS T3 column, using a gradient of 0.1% formic acid in water and 0.1% formic acid in acetonitrile. Mass spectrometric detection was performed on a Xevo G2-XS QTOF mass spectrometer, acquiring high-resolution data in both positive and negative ionization modes under MSe acquisition. Data were processed using dedicated software for peak picking, alignment, and internal standard correction, and metabolites were identified by matching against public databases. Data were processed using Progenesis QI software (Version 3.0, Waters Corporation, Milford, MA, USA) for peak picking, alignment, and internal standard correction. Metabolites were putatively identified by matching the acquired high-resolution MS/MS spectra and accurate mass against the Human Metabolome Database (HMDB) database.

### 2.10. Statistics Analysis

All results are expressed as mean ± SD. Statistical analysis was performed using GraphPad Prism 9 (San Diego, CA, USA). Differences between the two groups were analyzed using a two-tailed unpaired *t*-test. For comparisons among multiple groups, one-way ANOVA followed by Dunnett’s post hoc test was used. For microbial diversity, alpha diversity indices were also compared using one-way ANOVA with Dunnett’s test. A *p*-value ≤ 0.05 was considered statistically significant. Microbial α- and β-diversity indices were calculated using the QIIME2 pipeline and the vegan package in R. Amplicon sequence variants (ASVs) were generated with DADA2. β-diversity was assessed based on Bray–Curtis distance. Differences in α-diversity were compared by one-way ANOVA with Dunnett’s test. Differentially abundant microbial features between groups were identified using Linear discriminant analysis effect size (LEfSe) analysis, with the following parameters: a Kruskal–Wallis test significance threshold of 0.05, a logarithmic LDA score threshold of 2.0 for effect size, and no multiple-testing correction (method = “none”) as per the original implementation. Prior to analysis, data were normalized to relative abundances (sum to 1 per sample).

## 3. Results

### 3.1. Physiological Characteristics of B. coagulans In Vitro

**Figure 1:**
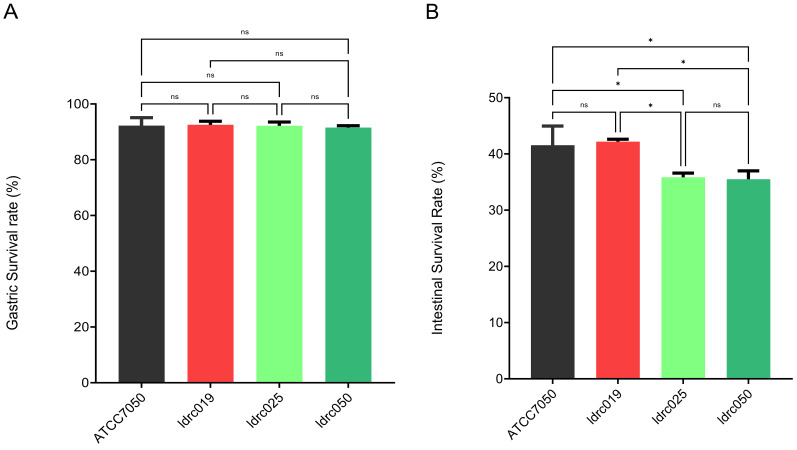
Survival and germination characteristics of *B. coagulans* strains under simulated gastrointestinal conditions. (**A**). Gastric survival rate. (**B**). intestinal germination rate. (*n* = 3 in (**A**,**B**), ns *p* > 0.05, * *p* < 0.05).

Prior screening identified *B. coagulans* idrc019 as exhibiting superior bile salt tolerance compared to other strains (Table S2). To further investigate the role of bile salt tolerance in the proliferation of *B. coagulans*, we selected both bile salt tolerant strains (*B. coagulans* idrc019 and *B. coagulans* ATCC 7050) and two randomly chosen bile salt intolerant strains (idrc025 and idrc050) from the pool of intolerant isolates for subsequent experiments. Under simulated gastrointestinal conditions, all tested strains showed high gastric resistance, with no significant differences between groups (Figure 1A). However, clear differences emerged when spore germination and proliferation were assessed under intestinal stress. Specifically, spores from bile salt-tolerant strains exhibited significantly higher post-germination survival than those from bile salt-intolerant strains (Figure 1B). These results support the hypothesis that bile salt tolerance in *B. coagulans* promotes spore germination and subsequent intestinal proliferation. Based on these findings, *B. coagulans* idrc019 was selected for further functional studies.

### 3.2. Evaluation of the Effects of Different Doses of B. coagulans idrc019 on Alleviating IBS

**Figure 2:**
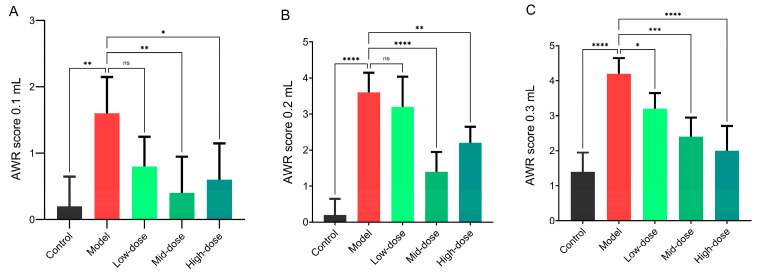
AWR scores in different groups at graded colorectal distension pressures: (**A**) 0.1 mL, (**B**) 0.2 mL, and (**C**) 0.3 mL. (*n* = 3 in (**A**–**C**), ns *p* > 0.05, * *p* < 0.05, ** *p* < 0.01, *** *p* < 0.001, **** *p* < 0.0001, vs. the Model group).

The effects of different doses of *B. coagulans* idrc019 on visceral sensitivity and inflammation in IBS model were tested in this study. Under varying intensities of colorectal distension (0.1 mL, 0.2 mL, and 0.3 mL), the strain exhibited differential improvement in visceral hypersensitivity. At the low-intensity stimulus (0.1 mL), the medium- and high-dose groups showed significantly lower abdominal withdrawal reflex scores compared to the model group, indicating effective alleviation of visceral pain. As the distension intensity increased, the protective effects became more distinct: at 0.2 mL, the medium- and high-dose groups, but not the Low-dose group, showed significantly better outcomes than the model group (*p* < 0.05, Figure 2B). At the highest stimulus intensity (0.3 mL), all treatment groups demonstrated significant alleviation of IBS symptoms compared to the model group (*p* < 0.05, Figure 2C). These fundings indicated that *B. coagulans* idrc019 exerts therapeutic effects in a dose-dependent manner

**Figure 3:**
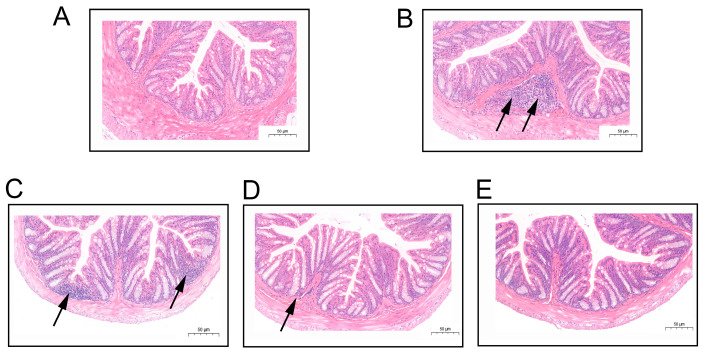
Histopathological analysis of mouse colon tissues. (**A**) Control group; (**B**) Model group; (**C**) Low-dose group; (**D**) Medium-dose group; (**E**) High-dose group. Black arrows indicate the presence of inflammatory cell infiltration at the marked locations. (magnification = 40×; scale bar = 50 µm).

The histopathological observations were further supported by semi-quantitative scoring. As shown in Table S3, the model group received a significantly elevated pathological score (2.3 ± 0.57), consistent with the observed focal lymphocytic infiltration (Figure 3B). Following low-dose intervention, the score decreased to 1.3 ± 0.57, corresponding to intact mucosal epithelium but persistent mild inflammatory infiltration (Figure 3C). In the medium- and high-dose groups, the scores were markedly reduced to 0.3 ± 0.57, aligning with the clear colonic structure, tightly arranged glands, abundant goblet cells, and absence of significant inflammatory lesions (Figure 3D,E). These quantitative results confirm the dose-dependent protective effect observed under microscopy.

In summary, our findings indicated that *B. coagulans* idrc019 exerts therapeutic effects in a dose-dependent manner. Under high-stress conditions, medium and high doses provide more stable and pronounced inhibition of visceral hypersensitivity, along with more significant anti-inflammatory effects.

### 3.3. Regulatory Effects of Different Doses of B. coagulans idrc019 on the Intestinal Immune System in Mice

**Figure 4:**
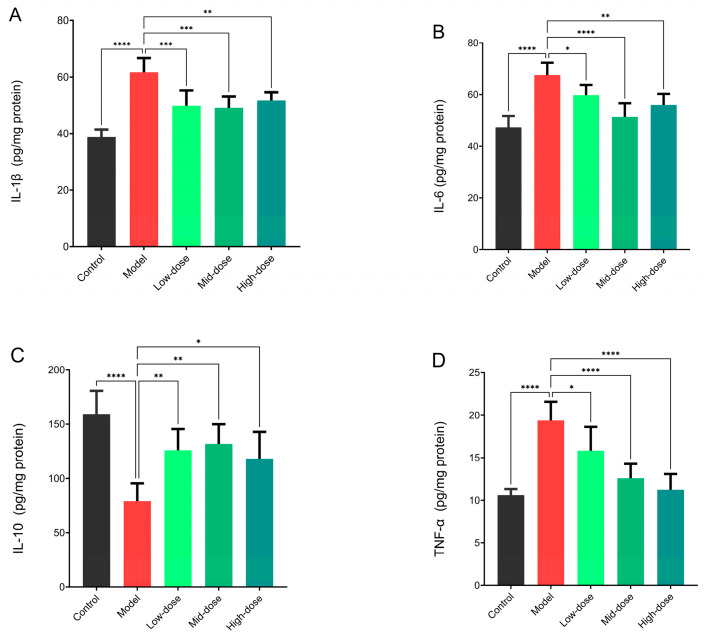
Expression levels of pro- and anti-inflammatory cytokines in colon tissue. (**A**) IL-1β, (**B**) IL-6, (**C**) IL-10, and (**D**) TNF-α. (*n* = 3 in (**A**–**D**), * *p* < 0.05, ** *p* < 0.01, *** *p* < 0.001, **** *p* < 0.0001, vs. the Model group).

Compared with the model group, different doses of *B. coagulans* idrc019 significantly reduced the expression levels of the pro-inflammatory cytokines IL-1β, IL-6, and TNF-α (*p* < 0.05, Figure 4). Additionally, compared with the model group, all dosage groups showed significantly elevated intestinal expression of the anti-inflammatory cytokine IL-10 (*p* < 0.05, Figure 4C). Moreover, compared with the control group, Occludin expression in the model group was significantly decreased (*p* < 0.05, Figure S1). Consistent with the trend observed for cytokines, supplementation with different doses of *B. coagulans* idrc019 significantly increased the expression level of Occludin protein in the mouse intestine (*p* < 0.05, Figure S1).

### 3.4. Regulatory Effects of Different Doses of B. coagulans idrc019 on Intestinal Mucosal Sensory Nerves

**Figure 5:**
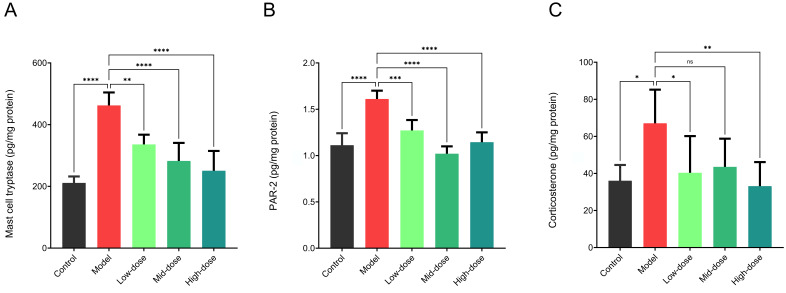
Levels of mast cell tryptase and PAR-2 in the colon and corticosterone in the serum. (**A**) Mast cell tryptase, (**B**) PAR-2, and (**C**) corticosterone were measured by ELISA. (*n* = 3 in (**A**–**C**), ns *p* > 0.05, * *p* < 0.05, ** *p* < 0.01, *** *p* < 0.001, **** *p* < 0.0001, vs. the Model group).

As shown in Figure 5, after intervention with different doses of *B. coagulans* idrc019, the expression levels of colonic mast cell tryptase and PAR-2 both decreased, indicating that *B. coagulans* idrc019 at different doses can effectively improve visceral hypersensitivity in mice (*p* < 0.05). Consistent with the above results, the expression levels of colonic mast cell tryptase and PAR-2 in the low-dose group were significantly higher than those in the medium- and high-dose groups, suggesting that the low-dose group was less effective than the high-dose groups in raising the pain threshold and alleviating visceral hypersensitivity (*p* < 0.05, Figure 5A,B). In addition, all dose groups of *B. coagulans* idrc019 showed significantly reduced serum corticosterone levels compared to the Model group, resulting in values that were numerically similar to the Control group (*p* < 0.05, Figure 5C).

### 3.5. Effects of Different Doses of B. coagulans idrc019 on Gut Microbiota Diversity and Composition

**Figure 6:**
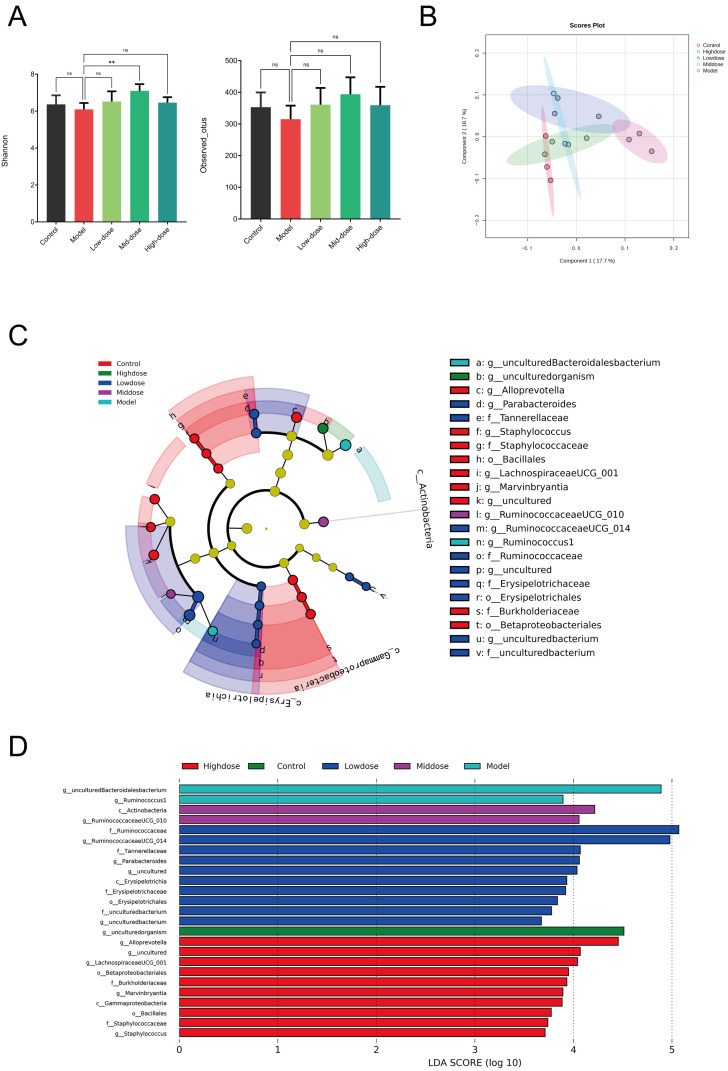
Effects of different dose of *B. coagulans* idrc019 on the gut microbiota diversity and composition. (**A**) Alpha diversity indices analysis (*n* = 5, ns *p* > 0.05, ** *p* < 0.01 vs. the Model group). (**B**) Beta diversity analysis. (**C**) LEfSe cladogram identified significant differential abundances of gut microbial taxa. (**D**) LDA effect size plot similarly visualized these taxonomic differences.

The microbiota analysis results are shown intake of different doses of *B. coagulans* idrc019 effectively restored the diversity of the mouse microbiota (Figure 6A). The absence of significant alpha-diversity differences may be due to limited sample size and high inter-individual variation. Following intervention, the composition of the intestinal microbiota in mice underwent dose-dependent changes (Figure 6B). LEfSe analysis revealed significant differences in gut microbial composition among the different experimental groups (Figure 6C). Based on linear discriminant analysis of differential genera, medium-dose supplementation of *B. coagulans* idrc019 helped restore the level of *Actinobacteria* in IBS mice (Figure 6D). In contrast, high-dose supplementation increased the relative abundance of the order *Bacillales* in the mouse intestine, suggesting that the probiotic may have promoted the growth or competitive advantage of this functional group through its metabolic activity or ecological interactions.

### 3.6. Effects of Different Doses of B. coagulans idrc019 on Fecal Metabolites

**Figure 7:**
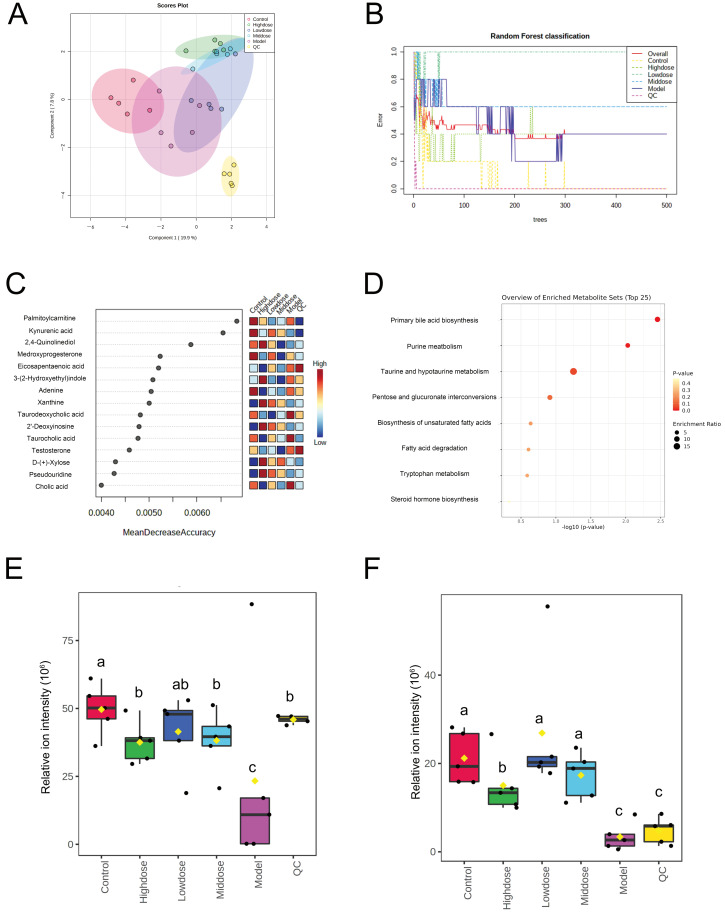
Metabolite profiling analysis in mice following treatment with different doses of *B. coagulans* idrc019. (**A**) principal component analysis. (**B**) Random forest classification. (**C**) Differential metabolites between groups. (**D**) Enrichment pathway analysis of metabolites; (**E**) relative abundance of kynurenic acid in mouse intestine. Different letters indicate statistically significant differences (*p* < 0.05). (**F**) relative abundance of testosterone in mouse intestine. Different letters indicate statistically significant differences (*p* < 0.05).

This study subsequently explored the effects of different doses of *B. coagulans* idrc019 on fecal metabolites in mice. Principal component analysis and random forest analysis revealed significant differences in metabolites between the control group and the other groups, indicating that the composition of fecal metabolites changed significantly after IBS modeling (Figure 7A,B). Random forest analysis further identified 14 differential metabolites between groups: palmitoylcarnitine, kynurenic acid, 4,6-dihydroxyquinoline, medroxyprogesterone, eicosapentaenoic acid, 3-(2-hydroxyethyl) indole, adenine, xanthine, taurodeoxycholic acid, 2′-deoxyinosine, taurocholic acid, testosterone, D-xylose, pseudouridine, and cholic acid (Figure 7C). Enrichment and pathway analysis of these metabolites showed that the screened differential metabolites were mainly involved in 8 metabolic pathways, closely related to maintaining host health (Figure 7D). Additionally, in this study, mice supplemented with different doses of idrc019 showed significantly increased fecal concentrations of kynurenine and testosterone compared to the model group (Figure 7E,F).

**Figure 8:**
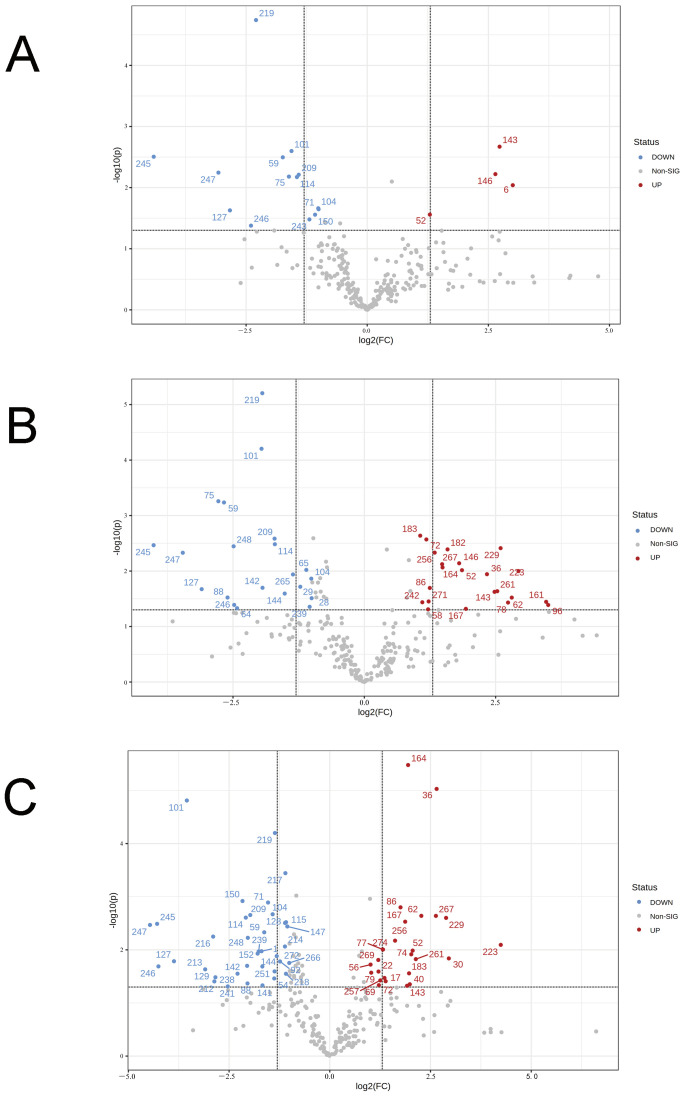
Volcano plot of intestinal metabolites in mice. (**A**) Low-dose *B. coagulans* idrc019 group vs. model group; (**B**) Medium-dose *B. coagulans* idrc019 group vs. model group; (**C**) High-dose *B. coagulans* idrc019 group vs. model group.

Subsequently, differential analysis of intestinal metabolites between the different dose probiotic treatment groups and the model group revealed that the regulatory capacity of *B. coagulans* idrc019 on mouse intestinal metabolites is dose-dependent (Figure 8). All differentially abundant metabolites and their corresponding user-defined (in-house) identifiers are provided in Supplementary Table S4. When a low dose of *B. coagulans* idrc019 was administered, only 18 metabolites changed significantly compared to the model group (including 14 downregulated and 4 upregulated). In contrast, with medium- and high-dose intake, 43 and 63 metabolites changed, respectively, compared to the model group. Therefore, the high-dose intake of *B. coagulans* may have a more pronounced effect on host intestinal metabolites.

## 4. Discussion

As a next-generation probiotic, *B. coagulans* possesses spores with high environmental stress resistance, ensuring high viability and stability in functional products [16]. Similar to most spore-forming bacteria, the spores of *B. coagulans* can germinate in the host duodenum and proliferate in the small intestine [17]. Following germination, the vegetative cells inevitably encounter bile salt stress. Therefore, the bile salt tolerance of the strain is closely associated with its survival and colonization capacity in the gut, representing a key physiological trait for exerting probiotic effects [18]. It is widely recognized that probiotics must be administered in adequate quantities as viable cells to confer clear health benefits to the host [19]. Accordingly, this study first evaluated the germination ability of the bile salt–tolerant strain *B. coagulans* irdc019 under simulated gastrointestinal conditions, confirming that its spores can tolerate multiple stressors such as gastric acid, bile salts, and pH fluctuations, and successfully germinate in intestinal environments, thereby providing a basis for subsequent probiotic function studies. Previous research has indicated that related strains of *B. coagulans* have ameliorative effects on IBS [9,20]. Based on this, the present study further employed an IBS animal model to systematically evaluate the therapeutic potential of *B. coagulans* idrc019.

The pathogenesis of IBS is complex, involving a combination of physiological and psychological factors that may contribute to disease onset. Currently, visceral hypersensitivity and mild inflammation are recognized as two fundamental clinical features of IBS, which can directly reflect disease severity in patients [21]. Thus, we initially assessed the efficacy of different doses of *B. coagulans* in alleviating IBS in mice by measuring the abdominal AWR score and evaluating colonic pathological changes. *B. coagulans* idrc019 ameliorated IBS-like symptoms, with a trend suggesting that the beneficial effects were more pronounced at medium and high doses. Under high-stress conditions, medium and high doses provide more stable and pronounced inhibition of visceral hypersensitivity, along with more significant anti-inflammatory effects. A recent study has reported that the efficacy of *B. coagulans* in alleviating constipation also follows a dose-dependent pattern, indicating that a sufficient quantity of the bacterium is required to exert its beneficial effects [22]. However, one limitation of this study is that colorectal distension stimulus was defined by balloon volume rather than direct intraluminal pressure measurement. Although the balloon was calibrated and the method is standard, altered colonic compliance in the model means that AWR scores may reflect both nociceptive and biomechanical changes. The observed volume-dependent responses nevertheless indicate that the intervention modulated visceral sensitivity, and future studies with pressure monitoring are warranted to distinguish these components.

In the mouse model of IBS, histopathological scoring and morphological observations confirmed that *C. rodentium* infection induced marked focal mononuclear inflammatory cell infiltration in the colon, which contributed to the pathological basis for subsequent visceral hypersensitivity. However, the specific subtypes of the infiltrating immune cells were not identified. Distinguishing between a chronic adaptive immune response dominated by T cells and an acute innate immune response primarily involving neutrophils is important for a deeper understanding of the evolution of the immune microenvironment in post-infectious IBS. In future work, we will further characterize the composition of the inflammatory cells through immunohistochemical staining or flow cytometry, in order to more precisely delineate the immunomodulatory targets involved in the protective effects of the bacterial intervention.

Moreover, the role of intestinal immune dysfunction and related cytokines has been extensively studied. Pro-inflammatory factors such as IL-6 and TNF-α can exacerbate inflammatory responses by promoting the survival of Th1/Th2 cells and inducing apoptosis of intestinal epithelial cells [23,24]. Although the specific mechanism of IL-1β in intestinal inflammation is not fully understood, its overexpression has been shown to stimulate T-cell proliferation, thereby upregulating the levels of IL-6 and TNF-α [25]. In contrast, the anti-inflammatory cytokine IL-10 alleviates inflammation by suppressing macrophage metabolic activity and downregulating pro-inflammatory factors produced by Th1 cells, B cells, macrophages, and natural killer cells [26]. This study further found that after intervention with *B. coagulans*, the level of IL-10 in the mouse colon was significantly increased compared with the model group (*p* < 0.05, Figure 4C), indicating that this strain can help restore intestinal immune balance by regulating multiple cytokines, including IL-6, TNF-α, IL-1β, and IL-10. This improvement in immune regulation may further contribute to maintaining the integrity of the intestinal epithelial barrier, which is primarily sustained by tight junction proteins such as the transmembrane proteins Occludin and Claudin, as well as the peripheral membrane protein ZO-1 [27]. Studies have indicated that prolonged exposure of intestinal epithelial cells to pro-inflammatory factors such as TNF-α and IL-6 in IBS can induce apoptosis, leading to epithelial barrier damage and low-grade inflammation [28,29]. In this study, the expression of Occludin in the colon of the model group was significantly lower than that of the control group (*p* < 0.05, Figure S1), while supplementation with *B. coagulans* restored its expression, suggesting that this strain may promote the repair of the intestinal barrier structure by mitigating inflammatory responses. It is noteworthy that the immunomodulatory effects of the probiotic exhibited dose-dependent, but not linearly correlated, patterns across different cytokines. Specifically, the low and medium doses appeared more effective in elevating the anti-inflammatory cytokine IL-10 and suppressing IL-1β. This may be related to a more favorable activation of regulatory immune cells or the priming of specific immune pathways at lower concentrations. In contrast, the suppression of TNF-α was more pronounced at medium and high doses, suggesting a stronger blockade of pro-inflammatory signaling. Furthermore, the medium dose showed a particularly strong inhibitory effect on IL-6, potentially indicating an optimal window for modulating the pro-/anti-inflammatory balance. These differential effects suggest that *B. coagulans* may coordinately regulate the immune response through multiple, dose-dependent targets. Future studies incorporating more detailed mechanistic investigations are warranted to elucidate the precise dose–response relationships.

Alterations in gut microbiota represent a critical component in the pathophysiology of IBS. The phylum *Actinobacteria*, which includes beneficial genera such as *Bifidobacterium*, contributes to intestinal homeostasis by producing short-chain fatty acids, modulating immunity, and reinforcing the epithelial barrier [30]. A reduction in its abundance, commonly observed in IBS, is linked to low-grade inflammation and visceral hypersensitivity. In this study, medium-dose *B. coagulans* idrc019 intervention restored *Actinobacteria* levels, suggesting a mechanism for its anti-inflammatory effects. Conversely, the order *Bacillales* plays a dual role: certain members exert probiotic effects through competitive exclusion and immunomodulation, while an elevated abundance may indicate dysbiosis or environmental stress [31]. Here, high-dose intervention increased *Bacillales* abundance, highlighting a dose-dependent shift in microbial structure. Collectively, *B. coagulans* may alleviate IBS symptoms in part by modulating key bacterial groups such as *Actinobacteria* and *Bacillales*, thereby promoting a more balanced gut microbiome.

In terms of metabolites, we observed that mice supplemented with different doses of idrc019 showed significantly increased fecal concentrations of kynurenine and testosterone compared to the model group (Figure 7E,F). Among the relevant metabolic pathways, kynurenine is closely linked to tryptophan metabolism. Growing evidence supports that tryptophan and its derivatives act as key regulators in maintaining neuro-emotional stability in IBS hosts, playing important roles in neurophysiology and immunology [32,33]. It has been reported that certain probiotics belonging to *Lactobacillus* and *Bifidobacterium* can modulate tryptophan metabolism by influencing the host kynurenine pathway, thereby affecting the gut–brain axis [34]. In the host, only a small fraction of tryptophan is used for synthesizing proteins, neurotransmitters (e.g., serotonin), and neuromodulators (e.g., tryptamine) [35]. Over 95% of tryptophan is degraded via the kynurenine pathway, yielding both neuroprotective compounds (such as kynurenic acid) and neurotoxic compounds (such as quinolinic acid) [36]. The present study indicates that intake of *B. coagulans* idrc019 exerts a similar effect in mice. Furthermore, steroid hormones, including sex hormones, have been reported to influence peripheral and central regulatory mechanisms of the gut–brain axis involved in IBS pathophysiology, leading to alterations in visceral sensitivity, motility, intestinal barrier function, and mucosal immune activation [37]. Testosterone, identified as a differentially abundant metabolite in this study, has been reported to exhibit analgesic effects in previous research. In rodent models, estrogen (estradiol) exacerbates stress-induced visceral hypersensitivity in males. Conversely, testosterone reduces visceral hypersensitivity in females [38]. Another study in male rats reported a negative correlation between testosterone and rectal sensation thresholds during balloon distension, suggesting a protective role of androgens [39]. Consistent with previous findings, the present study demonstrated that different doses of *B. coagulans* idrc0019 effectively elevated testosterone levels in mouse fecal metabolites, which may contribute to the reduction in visceral hypersensitivity in the host. Notably, palmitoylcarnitine emerged as a highly discriminatory metabolite in our analysis. Its alteration suggests a potential modulation of fatty acid β-oxidation or mitochondrial function in the context of IBS, which aligns with growing evidence linking metabolic shifts in energy utilization to gut–brain axis dysregulation [40].

It is worth noting that the *B. coagulans* idrc019 strain used in this study was isolated from healthy human feces (Table S2). This origin provides advantages for its probiotic candidacy: as a native gut isolate, it is pre-adapted to the gastrointestinal environment, thereby enhancing its survival and potential activity in mammalian models of IBS. Its source from a healthy donor also implies a precedent of safe coexistence within the complex human microbiome. Our data confirm that idrc019 tolerates simulated gastrointestinal conditions and alleviates disease symptoms in a mouse model without observed adverse effects, supporting its suitability as a probiotic. However, the small group size (n = 5) in this exploratory study limits statistical power. It was chosen based on common practice in similar models and ethical reduction principles. We therefore focus on reporting statistically significant, biologically clear effects. The consistent dose–response trends across multiple endpoints strengthen the main findings, which warrant confirmation in larger future studies.

## 5. Conclusions

This study systematically elucidated the dose-dependent efficacy and mechanisms of action of *B. coagulans* idrc019 in alleviating irritable bowel syndrome through in vitro and in vivo experiments. The findings revealed that the strain exhibits good bile salt tolerance, maintaining high survival and germination rates in a simulated gastrointestinal environment, thereby laying the foundation for its probiotic functionality in the intestines. In an IBS animal model, idrc019 significantly alleviated visceral hypersensitivity and colonic inflammation in a dose-dependent manner, with medium- and high-dose groups demonstrating more stable symptom improvement and anti-inflammatory effects, specifically by reducing levels of pro-inflammatory cytokines IL-1β, IL-6, and TNF-α while increasing the expression of the anti-inflammatory cytokine IL-10. Additionally, intervention with this strain enhanced the expression of the intestinal barrier protein Occludin, modulated gut microbiota composition by restoring the relative abundance of Actinobacteria, and influenced metabolic profiles, such as increasing levels of kynurenine and testosterone. These changes are closely associated with neuroimmune modulation and elevated pain thresholds. In summary, our data indicate that *B. coagulans* idrc019 intervention was associated with an amelioration of IBS pathological features. The observed improvements across multiple aspects, including trends in immune balance, gut microbiota composition, and metabolite profiles, collectively suggest a multi-faceted mode of action. These preliminary findings highlight the strain’s potential for therapeutic development, warranting further investigation in larger-scale studies to confirm and elaborate on its efficacy and mechanisms. Future research may focus on clinical translation and dosage standardization to advance the development and implementation of personalized probiotic therapies.

## Figures and Tables

**Table 1:** Animal Experimental Protocol.

Group	Dosage	Experimental Design
Control (*n* = 5)	Sterile saline (0.2 mL/d)	Sterile saline + LR + WAS + Sterile saline
Model (*n* = 5)	Sterile saline (0.2 mL/d)	*C. rodentium* + LR + WAS + Sterile saline
*B. coagulans* idrc019 Low dose (*n* = 5)	5 × 10^7^ CFU/mL (0.2 mL/d)	*C. rodentium* + LR + WAS + Low dose
*B. coagulans* idrc019 Medium dose (*n* = 5)	5 × 10^9^ CFU/mL (0.2 mL/d)	*C. rodentium* + LR + WAS + Medium dose
*B. coagulans* idrc019 High dose (*n* = 5)	5 × 10^11^ CFU/mL (0.2 mL/d)	*C. rodentium* + LR + WAS + High dose

Note: LR indicates intraperitoneal injection of lactated Ringer’s solution; WAS indicates water avoidance stress. Control group defined as healthy mice receiving no treatment, serving as the baseline control. Model group defined as mice infected with *C. rodentium* but not receiving subsequent experimental treatment, used to evaluate the pathological changes caused by the infection itself, serving as the positive pathological control for therapeutic interventions.

## Data Availability

The original contributions presented in this study are included in the article/Supplementary Material. Further inquiries can be directed to the corresponding author.

## Supplementary Materials

The following supporting information can be downloaded at: https://www.mdpi.com/article/10.3390/microorganisms14030701/s1, Table S1. Classification of *B. coagulans* strains according to their resistance to oxgall. Table S2. Description of the *B. coagulans* strains used in this study. Table S3. Colonic Pathological Scores. Table S4. Complete Metabolite List. Figure S1. The expression of Occludin in colon tissue. (*n* = 3, * *p* < 0.05, ** *p* < 0.01, **** *p* < 0.0001, vs. the Model group).
