# Supplementary material for: Bacillus coagulans idrc019 Attenuates Irritable Bowel Syndrome by Revealing Multimodal Protective Mechanisms
Source: Microorganisms. 2026 Mar 20;14(3):701. doi: 10.3390/microorganisms14030701 (PMC13029268; doi:10.3390/microorganisms14030701)
Supplement: Supplementary file 1 [file microorganisms-14-00701-s001.zip › Tables S1-S3, Figure S1.pdf]

microorganisms

MDPI

Supplementary Material

1 Supplementary Data

1.1 Supplementary Table

Table S1. Classification of *B. coagulans* strains according to their resistance to oxgall

	0.3 % oxgall		0.6 % oxgall	
Group	Bile resistant strains	Bile sensitive strains	Bile resistant strains	Bile sensitive strains
Delay of growth	40 < d <60 min	d ≥ 60 min	40 < d <60 min	d ≥ 60 min
LGG	+		+	
ATCC 7050	+		+	
ldrc001	+			+
ldrc002	+			+
ldrc003	+			+
ldrc004	+			+
ldrc005	+			+
ldrc006	+			+
ldrc007	+			+
ldrc008	+			+
ldrc009	+			+
ldrc010	+			+
ldrc011	+			+
ldrc012	+			+
ldrc013	+			+

ldrc014	+			+
ldrc015	+			+
ldrc016	+			+
ldrc017	+			+
ldrc018	+			+
ldrc019	+		+	
ldrc020	+			+
ldrc021	+			+
ldrc022	+			+
ldrc023	+			+
ldrc024	+			+
ldrc025		+		+
ldrc026		+		+
ldrc027		+		+
ldrc028		+		+
ldrc029		+		+
ldrc030		+		+
ldrc031		+		+
ldrc032		+		+
ldrc033		+		+
ldrc034		+		+
ldrc035		+		+
ldrc036		+		+

ldrc037	+	+
ldrc038	+	+
ldrc039	+	+
ldrc040	+	+
ldrc041	+	+
ldrc042	+	+
ldrc043	+	+
ldrc044	+	+
ldrc045	+	+
ldrc046	+	+
ldrc047	+	+
ldrc048	+	+
ldrc049	+	+
ldrc050	+	+

Note: "d": Delay of growth (in hours) between the control and oxgall bile cultures, noted in the "Remarks" column as a complementary indicator of tolerance. "+": Resistant to the oxgall. "-": Sensitive to the oxgall.

Table S2. Description of the *B. coagulans* strains used in this study

Strain ID	Sample ID	Site of Isolation	Source of Isolation	Year
idrc01	VZJSX1M1	Shaoxing, Zhejiang	Bok Choy	2024
idrc02	VZJSX1M2	Shaoxing, Zhejiang	Bok Choy	2024
idrc03	VZJNB1M1	Ningbo, Zhejiang	Bok Choy	2024
idrc04	VZJNB1M2	Ningbo, Zhejiang	Bok Choy	2024
idrc05	VZJJH1M1	Jinhua, Zhejiang	Bok Choy	2024
idrc06	VZJJH1M2	Jinhua, Zhejiang	Bok Choy	2024
idrc07	VZJHZ1M1	Hangzhou, Zhejiang	Bok Choy	2024
idrc08	VZJHZ1M2	Hangzhou, Zhejiang	Bok Choy	2024
idrc09	VSH1M1	Fushan Road, Pudong, Shanghai	Bok Choy	2024
idrc10	FSDZY1M1	Zhaoyuan, Shandong	Human feces	2024
idrc11	VSDZYM1M1	Zhaoyuan, Shandong	Bok Choy	2024
idrc12	VSDZZ1M1	Zaozhuang, Shandong	Bok Choy	2024
idrc13	VSDZZ1M2	Zaozhuang, Shandong	Bok Choy	2024
idrc14	VSDYT1M1	Yantai, Shandong	Bok Choy	2024
idrc15	VSDQD1M1	Qingdao, Shandong	Radish leaves	2024

idrc16	VSDLW1M1	Laiwu, Shandong	Bok Choy	2024
idrc17	VSDLW1M2	Laiwu, Shandong	Bok Choy	2024
idrc18	VSDJN1M1	Jining, Shandong	Bok Choy	2024
idrc19	FSDJN1M2	Jining, Shandong	Human feces	2024
idrc20	VSDJN1M1	Jinan, Shandong	Rapeseed	2024
idrc21	VSDBZ1M1	Binzhou, Shandong	Chinese cabbage	2024
idrc22	VSDBZ1M1	Binzhou, Shandong	Chinese cabbage	2024
idrc23	VJSYZ1M1	Yangzhou, Jiangsu	Bok Choy	2024
idrc24	VJSWX1M1	Wuxi, Jiangsu	Spinach	2024
idrc25	VJSWX2M1	Wuxi, Jiangsu	Bok Choy	2024
idrc26	VJSWX2M2	Wuxi, Jiangsu	Spinach	2024
idrc27	VJSWX2M3	Wuxi, Jiangsu	Bok Choy	2024
idrc28	VJSNJ3M1	Suburbs of Nanjing, Jiangsu	Bok Choy	2024
idrc29	VJSNJ4M1	Suburbs of Nanjing, Jiangsu	Bok Choy	2024
idrc30	VJSNJ5M1	Suburbs of Nanjing, Jiangsu	Bok Choy	2024
idrc31	VJSNJ6M1	Suburbs of Nanjing, Jiangsu	Bok Choy	2024

idrc32	VJSNJ7M1	Suburbs of Nanjing, Jiangsu	Bok Choy	2024
idrc33	VJSNJ3M1	Suburbs of Nanjing, Jiangsu	Bok Choy	2024
idrc34	VJSNJ4M1	Suburbs of Nanjing, Jiangsu	Bok Choy	2024
idrc35	VJSNJ5M1	Suburbs of Nanjing, Jiangsu	Bok Choy	2024
idrc36	VJSNJ6M1	Suburbs of Nanjing, Jiangsu	Bok Choy	2024
idrc37	VNJBGZ1M1	Bagua Zhou, Nanjing, Jiangsu	Spinach	2024
idrc38	VNJBGZ4M1	Bagua Zhou, Nanjing, Jiangsu	Bok Choy	2024
idrc39	VNJBGZ4M1	Bagua Zhou, Nanjing, Jiangsu	Bok Choy	2024
idrc40	VNJBGZ5M1	Bagua Zhou, Nanjing, Jiangsu	Rapeseed	2024
idrc41	VNJBGZ6M1	Bagua Zhou, Nanjing, Jiangsu	Winter crown daisy	2024
idrc42	VNJBGZ7M1	Bagua Zhou, Nanjing, Jiangsu	Bok Choy	2024
idrc43	VNJBGZ9M1	Bagua Zhou, Nanjing, Jiangsu	Rapeseed	2024
idrc44	VNJBGZ5M1	Bagua Zhou, Nanjing, Jiangsu	Rapeseed	2024
idrc46	VNJBGZ7M1	Bagua Zhou, Nanjing, Jiangsu	Bok Choy	2024
idrc47	VJSHYM1M1	Huai'an, Jiangsu	Lettuce	2024
idrc48	VJSHYM1M2	Huai'an, Jiangsu	Lettuce	2024

idrc49	VJSCZ1M1	Changzhou, Jiangsu	Bok Choy	2024
idrc50	VJSCZ1M2	Changzhou, Jiangsu	Bok Choy	2024

Table S3. Colonic Pathological Scores

Sample ID	Group	Pathological Score
A1	Control group	0 ± 0
A2	Model group	**2.3 ± 0.57**
A3	Low-dose group	**1.3 ± 0.57**
A4	Medium-dose group	**0.3 ±0.57**
A5	High-dose group	**0.3 ± 0.57**

1.2 Supplementary Figure

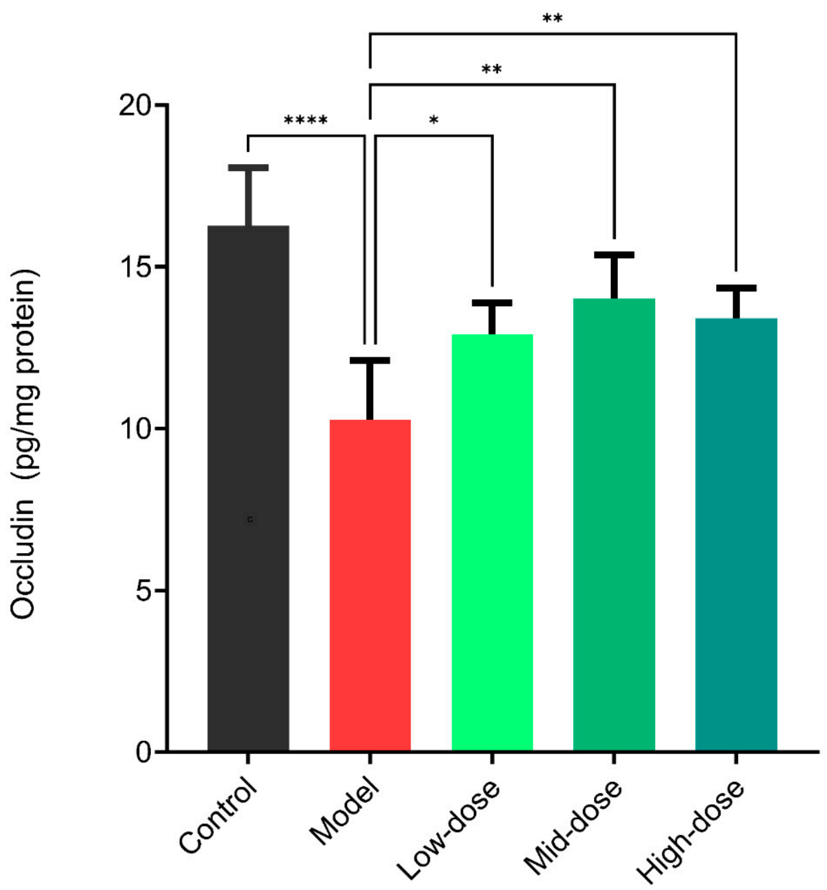

Figure S1. The expression of Occludin in colon tissue (*n*=3, **P* < 0.05, ** *P* < 0.01, **** *P* < 0.0001, *vs.* the Model group).
